# Gallstone ileus: report of two cases and a mini literature review

**DOI:** 10.1093/jscr/rjae588

**Published:** 2024-09-16

**Authors:** Georgios Gerasopoulos, Foteini Karagianni, Spyridon Nikas, Dimitrios Besikiaris, Kalliopi Veniadou, Maria Chondri, Panagiotis Routis, Sotiria Zonitsa, Despoina Sgouridi, Aggelos Karaklas

**Affiliations:** General Surgery Department, Imathia General Hospital, Veria Unit, Papagou Settlement, Veria 59132, Greece; General Surgery Department, Imathia General Hospital, Veria Unit, Papagou Settlement, Veria 59132, Greece; Diagnostic Radiology Department, Imathia General Hospital, Veria Unit, Papagou Settlement, Veria 59132, Greece; General Surgery Department, Imathia General Hospital, Veria Unit, Papagou Settlement, Veria 59132, Greece; Diagnostic Radiology Department, Imathia General Hospital, Veria Unit, Papagou Settlement, Veria 59132, Greece; General Surgery Department, Imathia General Hospital, Veria Unit, Papagou Settlement, Veria 59132, Greece; General Surgery Department, Volos General Hospital, Polymeri 134, Volos 38222, Greece; Diagnostic Radiology Department, Imathia General Hospital, Veria Unit, Papagou Settlement, Veria 59132, Greece; General Surgery Department, Imathia General Hospital, Veria Unit, Papagou Settlement, Veria 59132, Greece; General Surgery Department, Imathia General Hospital, Veria Unit, Papagou Settlement, Veria 59132, Greece; General Surgery Department, Attica General Hospital Sismanogleion-Amalia Fleming, Amalia Fleming Unit, Melissia, March 25th 14, Athens 15127, Greece

**Keywords:** gallstone ileus, small bowel obstruction, cholecysto-enteric fistula

## Abstract

Gallstone ileus is a relatively rare complication of cholelithiasis, and an uncommon cause of small bowel obstruction most commonly seen in elderly and debilitated people with associated comorbidities. Symptoms of gallstone ileus are insidious and may be vague while the delay in diagnosis results in a high mortality rate. Herein we report two cases of gallstone ileus in elderly patients with complex medical history who presented at the emergency department with abdominal pain and distension, vomiting and fluid/electrolyte disorders due to cholecysto-enteric fistula and bowel obstruction.

## Introduction

Gallstone ileus (GI) is a relatively rare complication of cholelithiasis and acute cholecystitis and is an infrequent cause of intestinal obstruction, accounting for 1%–4% of mechanical bowel obstructions [1]. GI are often characteristic of small bowel obstruction and include abdominal pain, distension, nausea, and vomiting [2] . Among imaging modalities, computed tomography (CT) is the diagnostic method of choice [3–5]. The treatment approach includes antibiotic therapy, fluid resuscitation and surgical procedure which can be achieved through simple emergency enterolithotomy, one-stage procedure including enterolithotomy, cholecystectomy, and fistula repair or two-stage surgical procedure involving an initial enterolithotomy as an emergency operation followed by cholecystectomy and fistula closure in 4–6 weeks later [1, 6]. We present two cases of GI as a consequence of cholecysto-pyloric and cholecysto-duodenal fistula, respectively.

## Case presentation

### Case 1

A 90-year-old male was transported by ambulance to the emergency department complaining of intermittent abdominal pain and vomiting, dehydration and mental disorientation started 3 days prior. The patient’s close relative reported a medical history of bedridden status in the past year, hypertension, type 2 diabetes mellitus, benign prostate hyperplasia, dyslipidemia, iron deficiency, and two past hospitalizations for acute cholecystitis. Upon arrival, he was afebrile (T: 37°C) and tachypneic (22 breaths/minute), and vital signs were the following: heart rate 110 bpm, blood pressure 125/85 mmHg, and oxygen saturation 94%. Examination of the abdomen revealed abdominal distension, hyperactive bowel sounds and mild left iliac fossa tenderness. Laboratory investigations revealed a leukocytosis of 10.990/mm^3^, hemoglobin of 12.4 g/dl, elevated levels of C-reactive protein of 11.17 mg/dl (N < 0,5 mg/dl), elevated serum urea and creatinine levels of 314 and 3.43 mg/dl respectively, whereas the rest of the laboratory parameters were within normal limits. A nasogastric feeding tube was placed with immediate drainage of 600 ml of intestinal fluids and intravenous fluid resuscitation was initiated promptly. Among the possible diagnoses, small bowel obstruction was prioritized due to clinical findings. To set the diagnosis, the patient underwent a CT scan with oral contrast only due to increased serum urea/creatinine levels. CT scan revealed an ambiguous image interpreted as possible small bowel intussusception at the jejunum–ileum transition with mesenteric swelling, gastric distension, swelling of the pylorus and small pleural effusions bilaterally.

**Figure 1:**
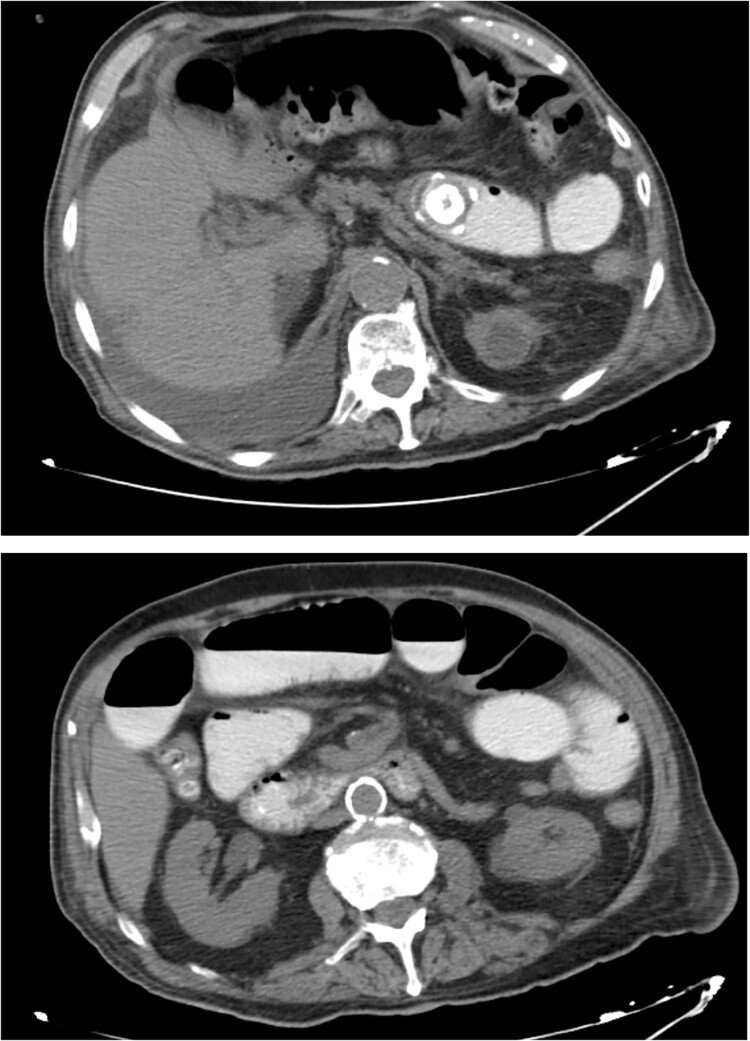
CT images of the abdomen revealed small bowel obstruction due to gallstone and presence of air and oral contrast into the gallbladder.

The patient was then admitted to the General Surgery department and treatment with broad-spectrum antibiotics (piperacillin/tazobactam and metronidazole) was initiated. On the second day of hospitalization, the patient underwent a repeat CT scan which indicated presence of air and oral contrast in the gallbladder, pneumobilia, swelling of the pylorus, and small bowel obstruction with the transition point being further into the ileum compared with the first CT scan (Fig. 1). Based on the comparison of the CT scan findings and past medical history of acute cholecystitis, gallstone ileus was established as the most possible diagnosis and the patient was led to the operating room.

**Figure 2:**
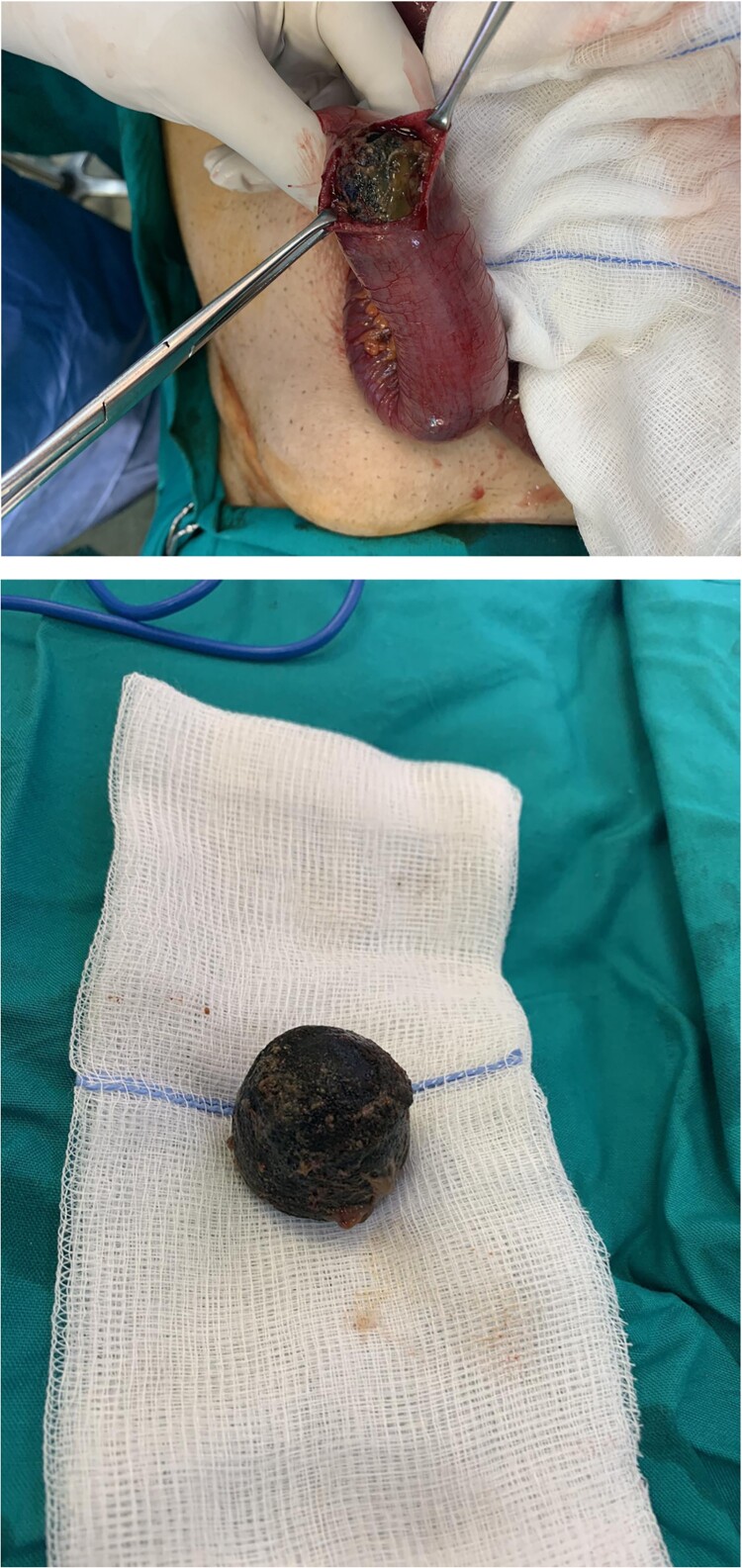
Intraoperative photo of a spherical gallstone of 3 cm in diameter extracted from an ileum intestinal loop.

The peritoneal cavity was exposed through a midline incision and ~300 ml of peritoneal fluid was drained. The dilated small intestine loops were retracted, and the obstruction point was located in the ileum, ~ 60 cm proximally to the ileocecal valve. After thorough inspection, there were no signs of strangulation on the obstructed loop and an enterolithotomy was performed. A large gallstone 3.5 cm in diameter was extracted and the enterotomy was sutured using absorbable sutures in two layers without signs of bowel ischemia or stenosis (Fig. 2). The peritoneal cavity was thoroughly irrigated, and two vacuum drains were placed.

Our patient showed a gradual clinical and laboratory improvement and on the third postoperative day the nasogastric tube was removed, followed by the removal of the surgical drains on the fourth postoperative day. On the seventh postoperative day, the laboratory tests revealed an improved value of serum creatinine of 2.1 mg/dl, and the patient was discharged following a nephrology consultation for outpatient treatment.

At the follow-ups (2 and 6 months later), the patient’s overall health was satisfactory with normal bowel function and no signs of recurrence.

### Case 2

**Figure 3:**
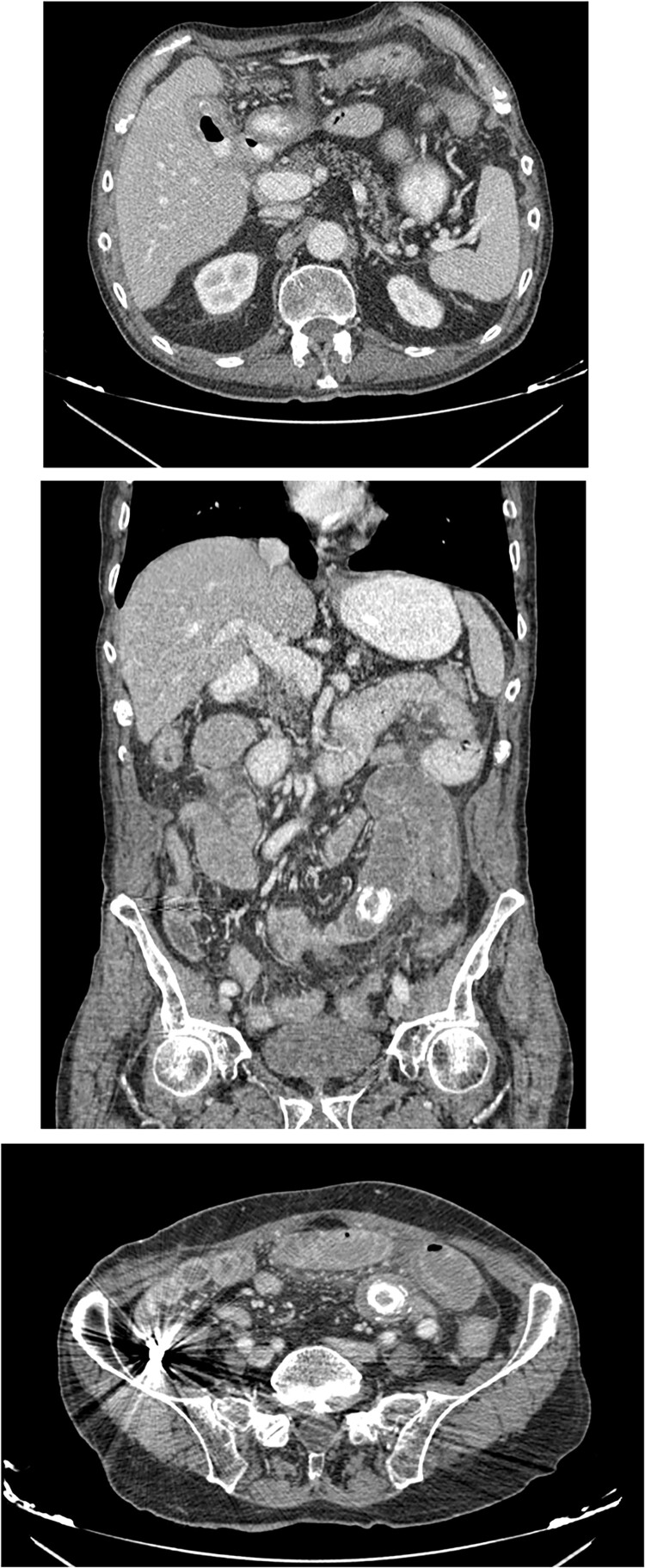
CT images with oral and IV contrast revealed presence of air and oral contrast in the gallbladder and small bowel obstruction caused by a mass in the jejunum–ileum transition attributed to a gallstone.

An 80-year-old male presented to the emergency department complaining of intermittent abdominal pain, vomiting and intense nausea beginning 3 days prior. The patient reported a medical history of type 2 diabetes mellitus, hypertension and coronary artery disease. Upon arrival, he was afebrile (T: 37°C) and tachypneic (24 breaths/minute), the following vital signs were: heart rate 98 bpm, blood pressure 160/95 mmHg, oxygen saturation 96%. Examination of the abdomen revealed significant hypoactive bowel sounds and diffuse tenderness. Laboratory investigations revealed a white blood cell count of 8.190/mm^3^, hemoglobin of 15.9 g/dl, and elevated levels of C - reactive protein of 15.94 mg/dl (N < 0.5 mg/dl) whereas the rest of the laboratory parameters were within normal limits. The patient initially underwent plain chest and abdomen radiographs which revealed small bowel gas-fluid levels indicating intestinal obstruction and intravenous fluids resuscitation was initiated. A CT scan with IV and oral contrast which was later performed revealed gastric, duodenal and small bowel loops distension and swelling caused by a high attenuation mass within the intestinal lumen at the jejunum-ileum transition which was attributed to a gallstone. Α second possible gallstone of smaller diameter was located in jejunum loops, and a cholecysto-duodenal fistula, of air and oral contrast in the gallbladder, pneumobilia, and intraperitoneal fluid in Douglas’s pouch were also present (Fig. 3).

The patient was then admitted to the General Surgery department and treatment with broad-spectrum antibiotics (piperacillin/tazobactam and metronidazole) was initiated.

**Figure 4:**
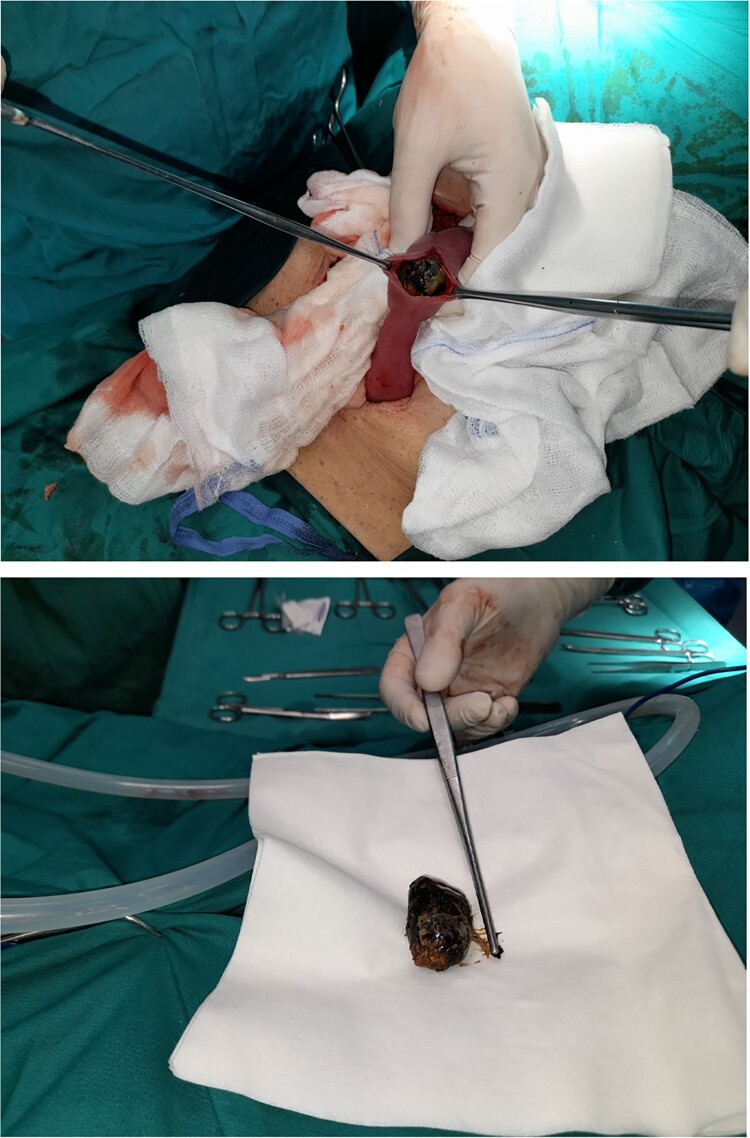
Intraoperative images of a 4 cm × 2 cm gallstone extracted from an ileum intestinal loop.

On the second day of hospitalization, as the diagnosis was clearly established, the patient was led to the operating room. The peritoneal cavity was exposed through a wide midline incision. The dilated small intestine loops were retracted, and the obstruction point was found at the jejunum-ileum transition. After thorough inspection, there were no signs of strangulation on the obstructed loop and enterolithotomy was performed. A gallstone with a size of 4 cm x 2 cm was extracted along with a second gallstone with a diameter of 1.5 cm located in a jejunum loop 10 cm proximally (Fig. 4). The enterotomy was sutured using absorbable sutures in two layers without signs of ischemia or stenosis. The peritoneal cavity was thoroughly irrigated, and two vacuum drains were placed.

Immediately postoperatively the patient was transferred to the Intensive Care Unit as extubating was impossible due to acid–base balance disorders, hemodynamic instability, and the patient’s increasing dependency on the ventilator.

During the patient’s hospitalization in the ICU, he developed an infiltrate-pneumonia of the right lower lobe the first postoperative day and on the second postoperative day the cultures from bronchoalveolar lavage, pharyngeal smear and rectum smear revealed microbial infection with *Klebsiella pneumoniae* as the predominant microorganism. On the third postoperative day the patient’s overall condition was deteriorating rapidly, with hemodynamic instability and increasing dependency on vasopressors, and on the fourth postoperative day the patient died in the ICU due to cardiac arrest.

## Discussion

GI is an uncommon complication of cho­lelithiasis and is defined as a mechanical intestinal obstruction due to impaction of one or more gallstones within the gastrointestinal tract [7]. GI occurs mostly in elderly patients [8, 9], in 0.15%–1.5% of cholelithiasis cases and accounts for <0.1% of ileus cases overall. The recurrence rate is 5%–8% [10], while only 50% of patients have a previous history of biliary pathology [11].

Pathophysiology of cholecysto-intestinal fistula emerges from acute cholecystitis where the inflammation in the gallbladder and surrounding structures leads to adhesion formation. All the above combined with pressure effect of the offending gallstone causes erosion through the gallbladder wall and leads to fistula formation between the gallbladder and the adjacent and adhered portion of the gastrointestinal tract, allowing gallstone passage [8, 12–14]. The proximal duodenum is most commonly affected, followed by the stomach, transverse colon, and other parts of the small bowel [8, 15]. Less common ways of entering the small bowel include through the common bile duct or a dilated ampulla of Vater [16] or post-endoscopic retrograde cholangiopancreatography (ERCP) [17, 18]. Once within the gastrointestinal lumen the gallstone proceeds distally and may pass spontaneously through the rectum, or it may become impacted and cause obstruction. The size of the gallstone, the site of fistula formation and bowel lumen will determine whether an impaction will occur. The majority of gallstones < 2–2.5 cm may pass spontaneously through the gastrointestinal tract and will be excreted in the stools while gallstones >5 cm are more likely to become impacted [7, 12, 13].

The most common impaction sites of the gallstone are the ileum (50.0%–60.5%), jejunum (16.1%–26.9%), duodenum (3.5%–14.6%), and colon (3.0%–4.1%) [1, 19], whereas less common locations include the stomach and upper duodenum [7, 12, 13, 20].

Most common symptoms of GI include nausea, vomiting, colic abdominal pain, and variable distension whereas acute cholecystitis and/or jaundice may be present in 10%–30% and <15%, respectively, of the patients at the time of bowel obstruction [8, 12, 20, 21]. Therefore, this entity is a diagnostic challenge because its symptoms are not exclusive and may be present in other pathologies that cause obstruction of the small intestine (adhesions, internal hernias, tumors, volvulus, etc.) [22].

Plain abdominal radiographs can be diagnostic of GI identifying Rigler’s triad, consisting of the presence of radiopaque stones (present in 10% of cases), pneumobilia (Gotta–Mentschler sign), and the distention of intestinal loops [23] with a sensitivity of 40%–70% [24]. Finally, CT with contrast is considered the diagnostic technique of choice, with sensitivity above 90% [3–5].

The treatment approach for GI consists of gastric decompression, antibiotic therapy, fluid/electrolyte resuscitation considering the dehydrated condition of these patients, and surgical intervention. Surgical treatment consists of emergency enterolithotomy alone, one-stage enterolithotomy with cholecystectomy/fistula closure and two-stage enterolithotomy with cholecystectomy/fistula closure after 4–6 weeks [1, 6]. One study indicated that mortality rate in patients who underwent enterotomy alone and patients who underwent the one-stage procedure was 11.7% and 16.9%, respectively [1], whereas 10% of patients with enterotomy alone have had recurrent biliary symptoms [7]. Moreover, persistence of cholecysto-intestinal fistula is a potential causal factor for retrograde cholecystitis or gallbladder cancer [25]. Urgent (one-stage) fistula repair is associated with significant postoperative complications as shown by a large study [9] with significantly longer hospitalization and higher mortality rate.

Based on those findings, two-stage surgery is considered the standard method for cases of gallstone ileus and those involving impaction at the small intestine [9], whereas one-stage surgery could be indicated for duodenal and colonic impaction [25].

In our cases, we treated two elderly patients of 90 and 80 years presenting with similar complaints (abdominal pain, distension, and vomiting) which, as revealed by the CT scan, were caused by GI. Considering their age, complex medical history, level of dehydration, and poor general preoperative condition we performed emergency open simple enterolithotomy on both patients. During the operations, the liver and gallbladder area was inspected laboriously due to multiple adhesions, thus significantly decreasing the success rate of a possible one-stage procedure on these patients, considering the aforementioned performance status. The impacted gallstones we extracted were spherical of 3.5 and 4 cm × 2 cm in diameter, respectively (and one non-obstructive of 1.5 cm in the second patient). The possibility of a cholecystectomy/fistula closure after 4–6 weeks was not offered to the first patient due to the co-morbidities and bedridden status. Αt the 6-month follow up no recurrent GI symptoms were present thus reinforcing the hypothesis of a natural fistula closure without further treatment.

## Conclusion

GI exhibits an insidious onset with vague signs and symptoms of intestinal obstruction. Accurate diagnosis is crucial, and surgeons should have a high clinical suspicion of this entity when history of acute/recurring cholecystitis is present. Considering the advanced age and comorbities of the majority of the patients presenting with this condition, relief of obstruction by enterolithotomy alone in the emergency setting is recommended, reserving the one-stage procedure for younger patients in good overall health and with absolute indication for biliary surgery.
